# Telomere Dynamics and Cortisol Response in Grazing Goats: Preliminary Insights Into Welfare Monitoring

**DOI:** 10.1002/age.70167

**Published:** 2026-07-29

**Authors:** Alessandra Iannuzzi, Ramona Pistucci, Maria Liccardo, Piera Iommelli, Raffaella Tudisco, Federico Infascelli, Fiorella Sarubbi

**Affiliations:** ^1^ Institute for Animal Production System in Mediterranean Environment National Research Council Portici Italy; ^2^ Dipartimento di Medicina di Precisione Università Degli Studi Della Campania Luigi Vanvitelli Napoli Italy; ^3^ Department of Experimental Medicine University of Rome Tor Vergata Rome Italy; ^4^ Department of Veterinary Medicine and Animal Production University of Napoli Federico II Naples Italy

**Keywords:** animal welfare biomarker, Camosciata delle Alpi goats, cortisol level, qPCR, telomere length

## Abstract

This study evaluated telomere length (TL) and serum cortisol concentration (SCC) as complementary stress biomarkers in grazing dairy goats. Twenty pluriparous “Camosciata delle Alpi” goats were monitored under extensive conditions at three 15‐day intervals. Milk‐derived DNA was used to assess TL by quantitative PCR; SCC was measured by enzyme immunoassay. Environmental parameters were recorded throughout the trial. SCC rose from 5.84 to 12.93 ng/mL at the second sampling, coinciding with adverse weather, and returned to baseline thereafter. TL decreased from 1.21 to 0.92 (telomere‐to‐single‐copy gene—T/S ratio) before recovering to 1.16. TL was negatively correlated with humidity and dew point, and positively correlated with rainfall, suggesting that moisture‐related stressors influence TL dynamics. These findings indicate that acute environmental challenges induce transient telomere shortening. Integrating TL and SCC provides a more comprehensive welfare assessment, with TL emerging as a promising non‐invasive genomic biomarker for stress and resilience in pasture‐based livestock systems.

Extensive grazing systems are widely adopted in marginal and mountainous areas, where they are generally associated with improved expression of natural behaviors and enhanced animal welfare by allowing greater freedom of movement, social interaction, and access to diverse botanical resources, these systems promote behavioral enrichment and physiological balance. Nevertheless, grazing animals are inevitably exposed to fluctuating and sometimes extreme environmental conditions—including temperature variability, humidity, precipitation, and sudden weather events—which may challenge physiological homeostasis and trigger stress‐related responses (FSA Panel on Animal Health and Welfare 2012).

There is growing interest in integrating molecular biomarkers into welfare assessment frameworks to complement traditional behavioral and physiological indicators. Among these, telomere length (TL) has emerged as a promising biomarker of oxidative stress, cellular aging, and overall physiological condition (Iannuzzi et al. 2023; Zhang et al. 2023). Telomeres are repetitive DNA sequences located at chromosome ends that maintain genomic stability and prevent chromosomal degradation (Nussey et al. 2013). Their progressive shortening with each cell division is further accelerated by oxidative stress and inflammatory processes (Von Zglinicki 2002). In livestock, TL has been associated with environmental conditions, physiological stress, and health status (Bateson 2016; Angelier et al. 2018), and validated across different biological matrices, including blood and milk in cattle (Iannuzzi et al. 2022). Telomere shortening has been linked to nutritional imbalance, disease, climatic stressors, and pollutant exposure (Iannuzzi et al. 2025), while TL maintenance has been associated with improved resilience and productive longevity. Despite this evidence, studies on telomere biology in small ruminants under real‐life farming conditions remain limited.

Cortisol, a widely used stress biomarker reflecting hypothalamic–pituitary–adrenal (HPA) axis activation (Ataallahi et al. 2022), provides a rapid and transient measure of acute stress, complementing TL which integrates cumulative cellular damage over time. Their combined evaluation may therefore offer insights into both immediate and long‐term physiological responses to environmental stressors.

The aim of this study was to evaluate the relationship between TL and serum cortisol concentration (SCC) in grazing goats exposed to natural environmental variability. We hypothesized that environmental stressors would induce concurrent changes in both biomarkers and that TL dynamics could reflect transient cellular responses to acute stress, potentially acting as a molecular “memory” of environmental challenges.

The study was carried out at a commercial farm in central Italy under extensive grazing conditions, in accordance with Directive 2010/63/EU and approved by the local Animal Ethics Committee (Protocol: PG/2019/0070006). Twenty lactating “Camosciata delle Alpi” goats in mid‐lactation were included in the trial. Animals grazed daily on botanically diverse natural pastures and received 700 g/head/day of a standardized concentrate composed of barley (23%), oats (22%), and faba beans (55%), with free access to water. Environmental conditions—including temperature, humidity, dew point, wind speed, atmospheric pressure, and precipitation—were continuously monitored throughout the experimental period. The chemical composition of both pasture and concentrate was determined prior to the trial according to Lo Presti et al. (2023).

The study was conducted from 1 April to 15 June 2021 under extensive grazing conditions. Sampling was performed at 15‐day intervals on 27 April, 11 May, and 25 May 2021 (Table 1). Blood samples were collected from the jugular vein, and SCC were determined using a solid‐phase competitive chemiluminescent enzyme immunoassay (Immulite 2000, Siemens). All samples were analyzed in a single batch to minimize inter‐assay variation. Milk samples were collected simultaneously for compositional analysis and DNA extraction. TL was assessed from milk‐derived DNA by qPCR using the telomere‐to‐single‐copy gene (T/S) ratio method (Cawthon 2009), with Pfaffl correction (Pfaffl 2001), following established guidelines (Lindrose et al. 2021). This non‐invasive approach has been validated as a reliable proxy for systemic telomere dynamics in dairy species (Iannuzzi et al. 2022). Statistical analyses were performed using SPSS v27; Pearson correlation coefficients assessed relationships among TL, SCC, and environmental parameters (*p* < 0.05).

**TABLE 1:** Physiological indicators, cellular stress markers, and microclimatic variables in experimental samples.

Samples	MY (kg/die)		Cortisol (ng/mL)		TL (T/S)		Dw (°C)		Humidity (%)		Rain (mm)	
	*μ*	SD	*μ*	SD	*μ*	SD	*μ*	SD	*μ*	SD	*μ*	SD
1	2.30	0.05	5.84	0.57	1.21	0.01	14.00	1.02	66.00	6.25	1.00	0.01
2	2.41	0.04	12.93	1.23	0.92	0.01	12.00	0.98	61.00	5.98	2.00	0.01
3	2.57	0.05	5.98	0.45	1.16	0.02	14.00	1.01	76.00	6.51	0.00	0.00

*Note:* Statistical significance was set at *p* < 0.05.

Abbreviations: Dw, dew point; MY, milk yield; TL, telomere length.

Milk production increased progressively from 2.30 to 2.57 L/head/day across the experimental period, likely reflecting enhanced nutritional intake from spring pasture, with no abrupt changes suggesting that productive performance was maintained despite environmental variability (Table 2). SCC rose markedly from 5.84 to 12.93 ng/mL at the second sampling, then returned to baseline (5.98 ng/mL), consistent with an acute stress response followed by recovery of physiological homeostasis (Table 1). The second sampling coincided with a short‐term adverse weather event characterized by severe thunderstorms, increased relative humidity (61%), a dew point of 12°C, and 2 mm of precipitation, representing the principal environmental stressor experienced by the animals during the study (Table 1). TL showed a parallel non‐linear pattern, decreasing from 1.21 to 0.92 (T/S ratio) before recovering to 1.16, suggesting a dynamic cellular response to environmental stress rather than simple linear attrition. Inter‐individual variability in TL increased during the second sampling period, with the coefficient of variation rising from 14.8% at T1 to 25.3% at T2, before decreasing to 16.5% at T3. This pattern suggests heterogeneous individual responses to the environmental challenge (Table 1; Supporting Information). We propose that the telomere shortening at the second sampling represents a “sentinel event” marking acute stress exposure, supported by the concurrent SCC peak indicating HPA axis activation. Environmental conditions during this period were characterized by increased humidity, elevated dew point, and severe thunderstorms (Table 1). Correlation analysis further supported this interpretation: TL was negatively correlated with humidity and dew point, suggesting that persistent moisture‐related conditions may contribute to cellular stress processes. A positive association was also observed with rainfall (*r* = 0.561, *p* < 0.001); however, this finding should be interpreted cautiously given the limited number of sampling points and the relatively low precipitation levels recorded during the study. No significant correlations were observed between TL and temperature or wind speed, indicating that specific environmental variables may differentially influence telomere dynamics.

**TABLE 2:** Correlation between cortisol level, telomeres, and meteorological data.

Sampling	Cortisol	Telomere
Production		
*r*	−0.202	−0.105
Sig.	NS	NS
Temperature		
*r*	−0.17	−0.080
Sig.	NS	0.648
Dew point		
*r*	−0.17	−0.487
Sig.	NS	0.003
Humidity		
*r*	−0.17	−0.522
Sig.	NS	0.001
Visibility		
*r*	−0.17	0.350
Sig.	NS	0.039
Wind		
*r*	−0.17	−0.145
Sig.	NS	0.404
Rain		
*r*	−0.17	0.561
Sig.	0.025	< 0.001
Cortisol		
*r*		0.047
Sig.		0.079
Telomere		
*r*	0.047	
Sig.	0.079	

Oxidative stress represents a key mechanism driving telomere shortening, as telomeric DNA is particularly vulnerable to reactive oxygen species (ROS)‐induced damage owing to its high guanine content (Von Zglinicki 2002). Extreme weather conditions can elevate ROS levels, thereby accelerating telomere erosion independently of cell division. Additionally, changes in milk cell composition may contribute to TL dynamics: during stress or inflammation, recruitment of immune cells with shorter telomeres may alter the telomere profile measured in milk‐derived DNA (Boutinaud and Jammes 2002; Effros et al. 2005). The TL recovery at the third sampling likely reflects compensatory mechanisms including reduced oxidative stress, cellular turnover, and selective apoptosis of critically short‐telomere cells.

Similar patterns of shortening followed by recovery have been reported in dairy cattle, where telomere attrition predicts productive lifespan (Seeker et al. 2021), and in wild animals showing seasonal TL variation (Chatelain et al. 2020; Fairlie et al. 2016). Although no direct statistical correlation was observed between SCC and TL, their coordinated temporal trends suggest complementary roles: SCC provides a rapid endocrine signal, while TL reflects cumulative cellular effects. Their integration may enhance the sensitivity and robustness of welfare assessment systems.

In conclusion, this study demonstrates that acute environmental stress is associated with transient telomere shortening and increased SCC in grazing goats. TL emerges as a sensitive, dynamic, and non‐invasive genomic biomarker for assessing stress and resilience in extensive production systems, whose integration with physiological indicators may contribute to more comprehensive and predictive welfare assessment tools. Exploring the genetic basis of TL variability, including its heritability and association with productive and health traits, represents a key area for future research.

## Author Contributions

**Alessandra Iannuzzi:** data curation, visualization, writing – original draft, methodology, supervision, writing – review and editing, validation, conceptualization, project administration. **Maria Liccardo:** investigation, formal analysis, writing – review and editing. **Fiorella Sarubbi:** conceptualization, data curation, writing – original draft, writing – review and editing, project administration, supervision, validation. **Raffaella Tudisco:** formal analysis, writing – review and editing, methodology, investigation, validation. **Federico Infascelli:** conceptualization, writing – review and editing, supervision. **Ramona Pistucci:** investigation, data curation, writing – review and editing, formal analysis. **Piera Iommelli:** data curation, visualization, writing – review and editing, methodology.

## Conflicts of Interest

The authors declare no conflicts of interest.

## Supporting information


**Data S1:** age70167‐sup‐0001‐supinfo.docx.

## Data Availability

The data used to support the findings of this study are included within the article.
