# Supplementary material for: Telomere Dynamics and Cortisol Response in Grazing Goats: Preliminary Insights Into Welfare Monitoring
Source: Anim Genet. 2026 Jul 29;57(4):e70167. doi: 10.1002/age.70167 (PMC13418842; doi:10.1002/age.70167)
Supplement: Supplementary file 1 — Data S1: age70167‐sup‐0001‐supinfo.docx. [file AGE-57-0-s001.docx]

**Supporting Information**

**Animals and diet**

The study was performed at a commercial farm in central Italy (400 m a.s.l.; 41°14'N, 13°50'E) under extensive grazing conditions, in accordance with Directive 2010/63/EU and approved by the local Animal Ethics Committee (Protocol: PG/2019/0070006). The region experiences an average annual rainfall of 530 mm and temperatures ranging between 6°C and 23°C. Daily meteorological data were recorded, including temperature, humidity, dew point, wind speed, atmospheric pressure (a.s.l.), and precipitation. Twenty lactating “Camosciata delle Alpi” goats (third lactation; body weight (BW) 45 ± 2.0 kg; days in milk (DIM) 60 ± 7were selected for the trial. All goats had ad libitum access to water and grazed daily on natural pasture (8:00 a.m. to 3:00 p.m. and 5:00 p.m. to 8:00 p.m.). The pasture consisted of a diverse botanical composition including *Trifolium alexandrinum*, *Vicia spp*., *Crataegus monogyna,* *Rubus ulmifolius*, *Clematis vitalba*, *Medicago sativa*, *Festuca arundinacea,* *Bromus catharticus*, and *Lolium perenne*. While housed indoors, goats received a concentrate supplement (700 g/head/day) composed of barley (23%), oats (22%), and faba beans (55%). Concentrate was determined by CP (20.77%), EE (2.12%), ash (12.19%), CF (22.24%), neutral detergent fibre, (NDF: 40.15%), acid detergent fibre (ADF:31.84%), and acid detergent lignin (ADL: 3.34%).

**sampling and TL analysis**

Sampling was conducted at fifteen-day intervals. Blood samples (10 mL) were collected from the jugular vein and centrifuged at 3500 rpm for 15 minutes. Serum was stored at -20°C and Serum Cortisol Concentration (SCC) was measured by competitive enzyme immunoassay (EIA) according to the manufacturer’s instructions. Milk yield was daily measured and before the onset of the experiment and milk samples (50 ml) were collected and kept at 4°C and brought in the laboratory for analysis. Milk chemical composition was analysed using Milko Scan 133B (Foss Matic, Hillerod, Denmark) standardized for goat milk.

10 ml of milk was used for genomic DNA extraction using the QIAamp Fast DNA Stool Mini Kit (QIAGEN) and assessed for quality (260/280 > 1.7, 260/230 > 1.8).

Telomere length was measured by qPCR using SYBR Green chemistry.

Briefly, the total reaction volume (10 μL) included SYBR Green Supermix (Bio-Rad), genomic DNA, and specific primers for telomere and *β-globin* as the single-copy gene (scg), following protocols by Cawthon (2009) for telomere and NCBI Reference Sequence: NC_056068.1 for scg. Each sample was run in triplicate (intra-assay) across three separate experiments (inter-assay), including a negative control (NTC).

The standard curve for each primer pair was used to assess amplification efficiency and linearity. The thermal cycling conditions were as follows: initial denaturation at 95 °C for 3 min, followed by 15 s at 95 °C and 15 s at 49 °C; then 35 cycles of 15 s at 94 °C, 40 s at 55 °C, and 30 s at 72 °C with signal acquisition. This was followed by 10 s at 80 °C and 15 s at 83 °C with signal acquisition. Melt curve analysis was performed by increasing the temperature from 60 to 95 °C in 0.5 °C increments, showing a specific peak at 75 °C for telomeres and 86.5 °C for the single-copy gene (scg).

qPCR amplification efficiencies were 97.6% for telomeres and 98.2% for the scg. Cq values showed high reproducibility, with a standard deviation below 0.5.

**Supplementary Table 1**: Inter-individual variability of telomere length (TL) in the 20 dairy goats at the three sampling times. Values are presented as mean telomere-to-single-copy gene ratio (T/S ratio), standard deviation (SD), and coefficient of variation (CV%).

| Sampling | Mean TL | | | SD | | CV (%) | |
| --- | --- | --- | --- | --- | --- | --- | --- |
| T1 | 1,21 |  |  | | 0,17 |  | 14,7 |
| T2 | 0,92 |  |  | | 0,22 |  | 24,5 |
| T3 | 1,16 |  |  | | 0,19 |  | 16,5 |
